# Forecasting upper respiratory tract infection burden using high-dimensional time series data and forecast combinations

**DOI:** 10.1371/journal.pcbi.1010892

**Published:** 2023-02-07

**Authors:** Jue Tao Lim, Kelvin Bryan Tan, John Abisheganaden, Borame L. Dickens

**Affiliations:** 1 Lee Kong Chian School of Medicine, Nanyang Technological University, Singapore; 2 Ministry of Health, Singapore; 3 Saw Swee Hock School of Public Health, National University of Singapore, Singapore; 4 Tan Tock Seng Hospital, Singapore; Yale School of Public Health, UNITED STATES

## Abstract

Upper respiratory tract infections (URTIs) represent a large strain on primary health resources. To mitigate URTI transmission and public health burdens, it is important to pre-empt and provide forward guidance on URTI burden, while taking into account various facets which influence URTI transmission. This is so that appropriate public health measures can be taken to mitigate strain on primary care resources. This study describes a new approach to forecasting URTIs which can be used for national public health resource planning. Specifically, using environmental and disease data comprising more than 1000 dimensions, we developed sub-models which optimizes model explainability, in-sample model fit, predictive accuracy and combines many weaker predictors over a 2-month time horizon to generate direct, point forecasts over a 1–8 week ahead forecast horizon. Predictive performance was evaluated using rolling out-of-sample forecast assessment within both periods with/without structural breaks in transmission over the period of 2012–2022. We showed that forecast combinations of 5 other forecasting models had better and more consistent predictive performance than other modelling approaches, over periods with and without structural breaks in transmission dynamics. Furthermore, epidemiological analysis on high dimensional data was enabled using post-selection inference, to show the dynamic association between lower temperature, increases in past relative humidity and absolute humidity and increased URTIs attendance. The methods proposed can be used for outbreak preparedness and guide healthcare resource planning, in both stable periods of transmission and periods where structural breaks in data occur.

## Author summary

Upper respiratory tract infections (URTIs) represent a large strain on primary health resources. To mitigate URTI transmission and public health burdens, it is important to pre-empt and provide forward guidance on URTI burden, while taking into account various facets which influence URTI transmission. This is so that appropriate public health measures can be taken to mitigate strain on primary care resources. This study describes a new approach to forecasting URTIs which can be used for national public health resource planning. Specifically, using a large number of environmental and disease data, we developed forecasting tools which optimize forecast performance to address this aim. Predictive performance was evaluated and we showed that our proposed method provides more consistent predictive performance than other approaches over different scenarios. Furthermore, we showed that lower temperature, increases in past relative humidity and absolute humidity were associated to increased URTIs attendance. The methods proposed can be used for outbreak preparedness and guide healthcare resource planning, in both stable periods of transmission and periods where structural breaks in data occur.

This is a *PLOS Computational Biology* Methods paper.

## Introduction

Upper Respiratory Tract Infections (URTIs) are usually characterised by irritation and swelling of the upper airways. They are caused by a variety of bacteria and viruses, and the infection can vary from a mild colds to life-threatening pneumonia [1]. Being one of the most common diseases, the global burden of URTI in 2019 is estimated to be 17.2 billion [2]. Although a majority of individuals contracting URTIs present mild symptoms, the estimated economic burden of non-influenza related viral URTIs in the United States alone is estimated to be 22.5 billion USD [3] and an estimated 2 billion USD is spent on over-the-counter treatments for URTIs [4].

URTIs are endemic in Singapore and has resulted in over ~2400 daily visits on average in polyclinics between 2012–2022, thereby representing a significant strain on primary health resources. On an individual level, risk factors include smoking, a history of allergic disorders, close contact with children, among many others [1]. Past studies also delineated the association between cold climates and URTI [5], with differential severity across different age subgroups [6]. In other studies, URTI was negatively associated to relative [7,8], absolute humidity [9] and increasing hours in sunshine [10]. To mitigate URTI transmission and public health burdens, it is important to pre-empt and provide forward guidance on URTI burden, while taking into account various facets which influence URTI transmission. This is so that appropriate public health measures can be taken to mitigate strain on primary care resources.

Statistical approaches to forecast URTI average daily attendances need to meet certain criterion to be of use to policymakers, namely **a)** able to generate forecasts using data available only up to the time a forecast is made; **b)** can forecast at a horizon to give sufficient lead time for public health responses; **c)** have good and validated predictive performance using data that was not used in its construction and **d)** be able to rapidly generate forecasts. While transmission dynamic modelling exploits epidemiological knowledge to model what-if scenarios and potential disease interventions–such as movement restrictions or face coverings and may be useful for predicting long-term changes to epidemic dynamics caused by changing herd immunity levels, climate change or by the age structure of a population [11]. A large limitation is the difficulty in integrating and calibrating transmission dynamic models to real-time data streams, such as meteorological or surveillance data [11]. In contrast, more reductionist statistical approaches which describe the observations rather than the underlying process can be well suited to integration with multiple live data streams and can provide good forecast accuracy if future conditions do not stray too far from the data used to parameterize them.

Frameworks have been developed to forecast URTIs and other respiratory illnesses. These comprise of and are not limited to statistical models using meteorological variables, including panel generalized linear models and generalized additive models [12], machine learning methods such as the Long Short-Term Memory neural network and multilayer perceptron which incorporates both meteorological and ambient air pollutant data [13,14], transmission dynamic models incorporating epidemiological information [15] and a combination of these forecasting approaches as a forecasting ensemble by weighing forecasts generated from different models by their past forecast performance [16]. Although the aforementioned models meet many of the criteria noted above, it is noteworthy that many have been developed in locations where transmission is seasonal with minimal assessment on forecasting skill. This suggests that they may not be applicable to locations where respiratory disease transmission is persistent all year-round with the forecast skill of proposed models not rigorously proven to be superior to any baseline alternatives. Incorporation of disease data other than that of lagged values of the predictor itself has not been substantially explored in forecasting studies, even though the incidence of different diseases may provide information on facets of URTIs transmission, and proxy for mobility or social mixing related factors leading to increased transmission. These models have also not been assessed in periods where structural breaks occur–where the transmission patterns of disease undergo substantial change, such as in periods where there are drastic changes in social mixing or movement.

Therefore, in this paper, we describe a new approach to forecasting URTIs which can be used for public health resource planning in Singapore. In contrast to previous studies, we explored and assessed the utility of our approach in forecasting URTIs in an URTI endemic setting where transmission occurs year-round. Furthermore, we assessed our methods in periods with and without structural breaks in transmission by incorporating pre and post COVID-19 pandemic time series data to assess forecast stability. By incorporating a high-dimensional set of disease data which comprises over 40 disease time series as well as environmental covariates, our models account for temporal co-movement of disease transmission. The model specifically optimizes predictive accuracy over a 2-month time horizon with predictive performance evaluated using out-of-sample forecast assessment within both periods with/without structural breaks in transmission. We demonstrate that this approach enables the generation of quick and accurate forecasts, and allows easy epidemiological interpretation of the effects of environmental covariates on URTI transmission, in contrast to more sophisticated ensemble and deep-learning models commonly employed in machine learning literature. Using an average of 5 other forecasting models incorporating over 280 dimensions, we show that our approach has better and more consistent predictive performance than other modelling approaches.

## Materials and methods

### Disease surveillance data

The average daily polyclinic attendances of acute upper respiratory tract infections, conjunctivitis, Diarrhea, chickenpox and Hand, Foot and Mouth Disease is collected by the Ministry of Health, Singapore and reported for each epidemiological week. Diseases where case counts were consistently reported to be zero were removed from the analysis (See S1 Text for full list of data and data sources). Disease surveillance data was reported from Epidemiological Week (EW) 1 of 2012 –EW 32 of 2022 and was publicly available on the weekly infectious disease bulletin, published by the Ministry of Health, Singapore [17].

### Methods: Environmental data

Climate data was obtained from ERA5-Land, published by the European Centre for Medium-Range Weather Forecasts. ERA5-Land provides hourly estimates across a 30km grid, which we have aggregated to the epidemiological week timescale and spatially averaged over Singapore. Mean, minimum and maximum air temperature at 2m (Kelvin) was calculated to represent thermal forcing and stress on host populations, and total rainfall (metres) summated for each epiweek to proxy for its influence on population mixing behavior and time spent outdoors. Air temperature and dewpoint temperature were utilized to calculate saturation vapor pressure (kPa), actual vapor pressure (kPa) [18], relative humidity (%) and absolute humidity (g/m^3^) using standard formula [19]. The leaf area index was also utilised to represent greenness in the two categories of high and low vegetation where a value of zero represents bare ground. The former represents evergreen trees, deciduous trees, mixed forest/woodland and interrupted forest. The latter consists of crops, mixed farming, grasses and shrubs.

### Generating point forecasts through individual models and forecast combinations

We aim to predict URTI polyclinic attendances for 1–8 weeks ahead. Predictors here include 1–8 weeks lag of all other reported disease case counts as well as environmental variables reported in the data sources described above. Multiple lags were incorporated into our forecast to serve as additional predictors to characterize the temporal dynamics of the dependent variable of interest. These comprise of a high-dimensional set of covariates (>280) used to forecast our dependent variable of interest. Here, standard methods for forecasting which include likelihood-based methods, will likely suffer from overfitting, high prediction variance and consequentially poor forecasts. Machine learning methods incorporating regularization and ensemble-based methods were therefore the primary tools proposed for forecasting in this study although the former’s performance was also explored.

Namely, we consider the following conditional expectation E(.|.) as the *h*-week ahead forecast for URTI average daily attendances:

$$E(y_{t+h,-D}|X,\beta )=\beta_{0}+\sum_{l=L_{-D}\in \{0,1,2\ldots \}}{\beta_{l,y}y}_{t-l,-D}+\sum_{d\in D}^{D}\sum_{{l=L}_{D}\in \{0,1,2\ldots \}}\beta_{l,d,y}y_{t-l,d}+\sum_{e}^{E}\sum_{{l=L}_{E}\in \{0,1,2\ldots \}}\beta_{l,e,y}x_{t-l,e}$$ (1)

where *t* denotes the contemporaneous time point, *y*_*t*+*h*,−*D*_ denotes URTI average daily attendances at *t*+*h*. Note here that we denote *D* as the set of diseases considered to be predictors asides from URTI, and −*D* the index for average URTI daily attendances. As explanatory variables, the structural form is similar to an autoregressive model with exogenous variables. Autoregressive terms include past and contemporaneous observations of URTI average daily attendances *y*_*t*−*l*,−*D*_, where *l* denotes the lag order up to a maximum of *L*_*D*_ lags. Transmission of a disease of interest may be partially also explained by other diseases such as mobility or social mixing related factors. Therefore, additional predictors for URTI include other communicable diseases reported on the infectious disease bulletin *y*_*t*−*l*,*d*_, *d*∈*D*. Furthermore, environmental forcing on future disease burden is incorporated through climate covariates *x*_*t*−*l*,*e*_, where *e* denotes the climatic variable. We assumed that *y*_*t*+*h*,−*D*_ is normally distributed so that the conditional mean of *y*_*t*+*h*,−*D*_ is the *h*-week ahead forecast. Only direct forecasts were considered for the study due to the inclusion of exogenous environmental variables as predictors.

We considered 2 regularization strategies here to estimate the parameters ***β***∈{*β*_0_, *β*_*l*,*y*_, *β*_*l*,*d*_, *β*_*l*,*e*_} the number of lags {*L*_−*D*_, *L*_*D*_, *L*_*E*_} and the set of parameters {−*D*,*D*,*E*}. First, the Least Absolute Shrinkage and Selection Operator (LASSO) framework extends standard regression methods through inducing variable sparsity by simultaneously selecting which parameters to include in the model and what their values should be. The LASSO framework has the following objective function, which finds the optimal set of parameters ***β*** by optimizing:

$$\underset{\beta }{\mathrm{argmin}}{\left(y-X\beta \right)}^{2}+\lambda_{1}{\left|\left|\beta \right|\right|}_{1}$$ (2)

which is the total squared difference between the dependent variable *y*_*i*_ and the multiple between the predictor matrix **X**′ = {**y**_***t***−***l***,−***D***_, **y**_***t***−***l***,***d***_, **x**_***t***−***l***_} and coefficients ***β***′ = {***β***_***l*,*y***_, ***β***_***l*,*d*,*y***_, ***β***_***l*,*e*,*y***_}, as well as a penalty term controlled by an additional parameter λ_1_, which controls model complexity.

We also considered the elastic net penalty to overcome limitations related to LASSO, such as poor variable selection among a group of highly correlated variables. This may occur for the case of selecting climate variables in determining disease burden, due to their tendency to co-move. The elastic net adds a quadratic component λ_2_||***β***||^2^ to the penalty to combine the benefits of LASSO and ridge regression, for which the latter is mainly used to alleviate the issue of estimation among highly correlated variables:

$$\underset{\beta }{\mathrm{argmin}}{\left(y-X\beta \right)}^{2}{+\lambda_{1}{\left|\left|\beta \right|\right|}_{1}+\lambda }_{2}{\left|\left|\beta \right|\right|}^{2}.$$ (3)

Where the optimal tuning parameters λ_1_, λ_2_ for both (2) and (3), were obtained through 10-fold cross-validation in the training dataset, which has less bias in estimating the cross-validation error versus alternatives, such as leave-one-out or 5-fold cross-validation [20]. Based on the value of the optimal tuning parameters, automatic variable selection is performed for both elastic net and LASSO by forcing irrelevant variables to zero.

Asides from regularization, we also explored using gradient boosting, an ensemble method, which provides direct forecasts by combining the predictions of many weak predictors into a final prediction model. The weak predictors were taken here as regression trees, which were iteratively fitted to the data. Briefly, in the initial stage, a singular decision tree was fitted to the dependent variable, taken as *h*-week ahead of URTI average daily attendances. The subsequent trees were fitted to the discrepancy between the predictions generated by the previous tree and actual observations. This was continued until the discrepancy between predictions and data crossed a pre-specified threshold. In all iterations, regression trees were using the predictors as described in (1).

Lastly, a baseline linear model was considered with the only disease case counts of interest as predictors. Environmental covariates and other diseases were not included due to the tendency for linear models to overfit data and have high predictive variance. The number of lags for a maximum of 8 lags were selected using backward stepwise selection with the Akaike Information Criterion being taken as the metric to remove a variable. The *h*-week ahead conditional forecast was then taken as:

$$E(y_{t+h,-D}|X,\beta )=\beta_{0}+\sum_{L_{-D}\in \{0,1,2\ldots \}}{\beta_{l,y}y}_{t-l,-D}$$ (4)

with regression coefficients *β*_0_, *β*_*l*,*y*_ estimated through least squares.

### Forecast assessment

First, we split the initial training data set to 60% of all available data. This comprises of observations from the first time point where disease surveillance data was collected to the point where 60% of observations were collected. From that point onwards, in a rolling manner, *h*-week ahead direct forecasts were generated with *h* separate sub-models trained using the model specification in (1) and (4) and the 4 models described above. Separately, we also considered: (**a**) a forecast ensemble, taken as the simple average of the forecast generated by the 4 models and (**b**) a naïve forecast where the latest available URTI attendance observation equates to the 1-step ahead prediction. These were used as additional forecasts to be assessed against.

For each additional epidemiological week, an additional week of observation was included into the forecasting models where each *h*-week ahead sub-model was retrained and the *h*-week ahead conditional forecast regenerated. This strategy ensures that no future data is incorporated in the forecast generated in the contemporaneous time-step. The actual observations were then compared post-hoc against the forecasts with forecast performance summarized into 4 key summary statistics, namely: (**a**) mean absolute forecast error (**b**) root mean-squared forecast error (**c**) mean absolute percentage forecast error and (**d**) mean absolute scale error. The summary statistics (**a**) and (**b**) summarise the average amount of error each forecasting model makes versus the actual observations, (**c**) summarises the percentage error each forecasting model makes versus the actual observations and (**d**) compares whether the absolute forecast error that each forecasting model makes is more or less than the naïve one-step ahead forecast in a pairwise fashion between models. In addition, the Diebold-Mariano hypothesis test was conducted pairwise between models to statistically ascertain the equivalence or non-equivalence between forecasts [21].

### Understanding effects of meteorological variables on URTI average daily attendances

Aside from generating forecasts, the LASSO and linear model can be employed to understand how environmental factors contribute to increased or decreased disease burden over time. The LASSO was chosen due to the ease of interpreting coefficients due to the linear specification. We first trained the LASSO model on the specification (1) on the full dataset, finding the optimal tuning parameter using 10-fold cross-validation. Confidence intervals for each regression coefficient were obtained through post-selection inference [22], namely by training a linear model using only covariates selected through the LASSO procedure. These coefficients then provided the expected increase or decrease in URTI average daily attendances for a number of weeks ahead, given a contemporaneous or past unit rise in a model covariate.

## Results

### Disease surveillance and meteorological data

**Fig 1:**
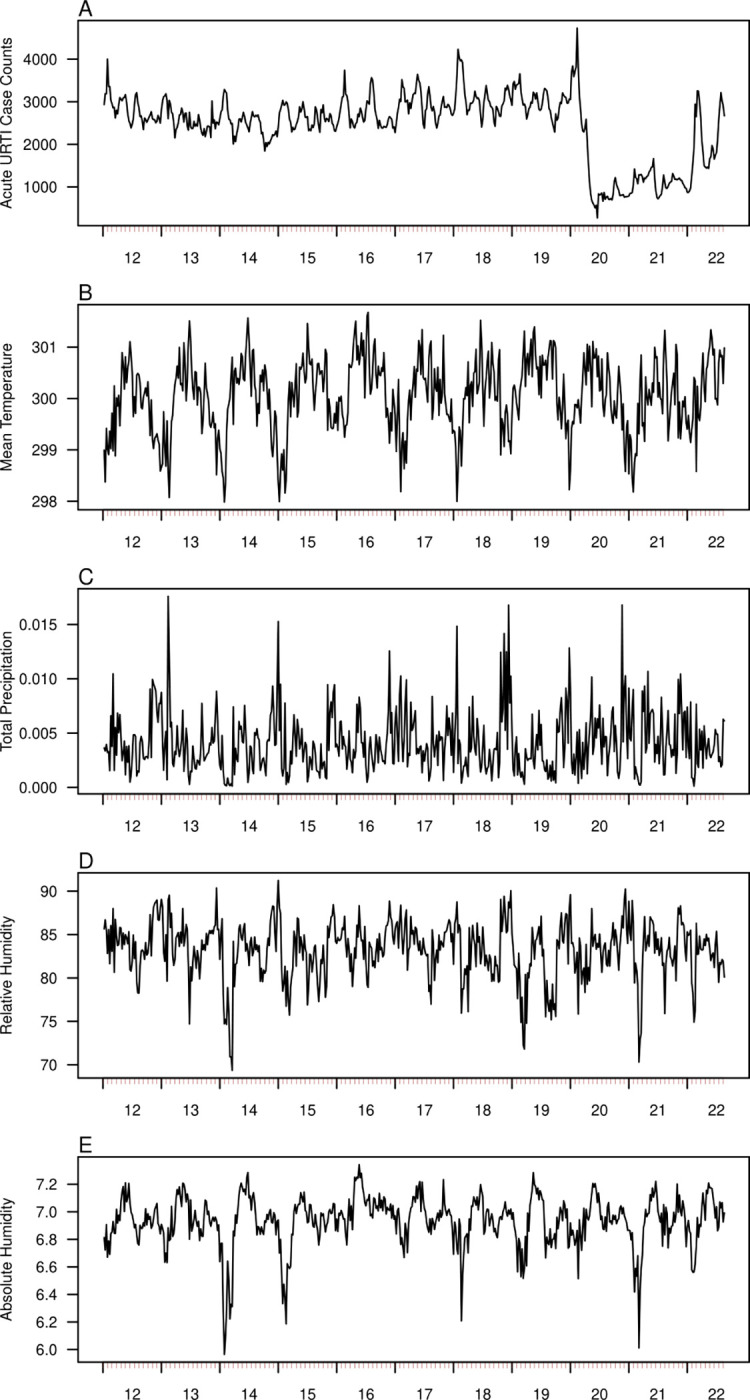
Weekly time series data from epiweek 1 2012 to epiweek 35 2022 on (A) average daily polyclinic attendances for upper respiratory tract infections (URTIs), (B) mean temperature (Kelvin), (C) mean total precipitation (Metres), (D) mean relative humidity (%) and (E) mean absolute humidity (in g/m^3^)].

We utilised data on URTI polyclinic attendances (Fig 1A), temperature (Fig 1B), total precipitation (Fig 1C), relative (Fig 1D) and absolute humidity (Fig 1E). Disease surveillance data seem to vary with no clear pattern from the period of 2012–2020, but with the deployment of non-pharmaceutical interventions due to the COVID-19 pandemic around early 2020, a large dip in the number of polyclinic attendances was observed which gradually reverted to pre-pandemic values in 2022 (Fig 1A). Meteorological variables were also relatively constant with an average value of 300.061K (Fig 1B, Range: 297.982K – 301.680K), 0.004m (Fig 1C, Range: 0.000m – 0.018m), 6.935% (Fig 1D, Range: 5.964%– 7.343%) and 83.204g/m^3^ (Fig 1E, Range: 69.356g/m^3^–91.239g/m^3^) for temperature, total precipitation, relative and absolute humidity respectively.

### Overall forecasting performance for URTI attendances for 1 to 8 weeks ahead

Model calibration was conducted using covariates for up to 8-week lags as described in the model specification (1) and (4) in the Materials and Methods section. Forecast combinations and naïve forecasts were also taken as separate forecasts to be considered. A total of 8 sets of models were trained for each respective forecasting window using data up until the point of forecast. The forecasts showed relatively good concordance with actual data up to one month ahead but tended to mean-revert at longer forecast horizons. We present the forecast for each forecasting model plotted against actual observations for each specific time horizon over 2018–2022 in the Supplementary Information.

**Fig 2:**
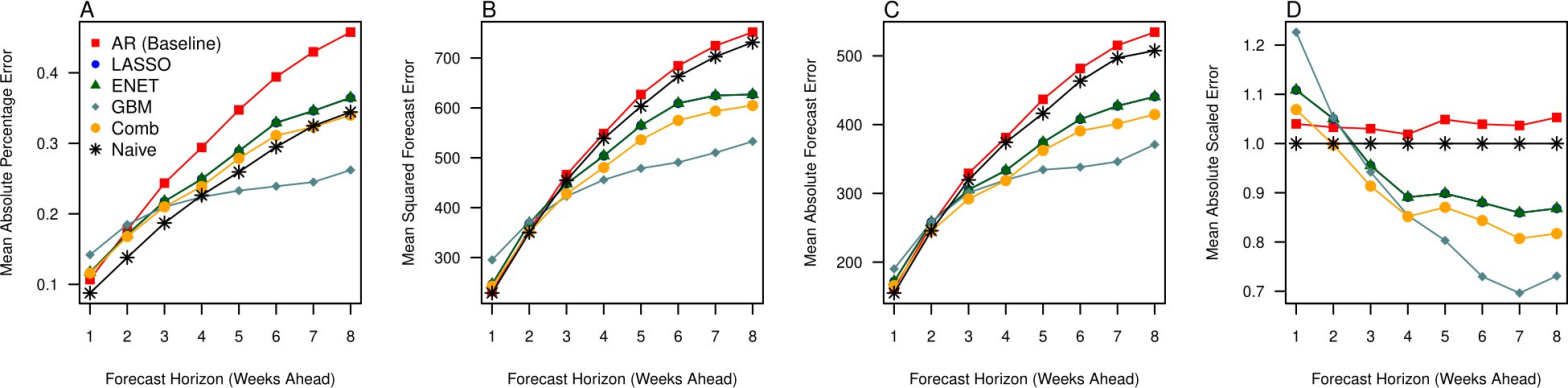
Forecast assessment statistics across the forecast dataset, comparing observations in the period of 2018–2022 to the 6 forecasting models, which includes the baseline autoregressive (AR) model, the Least Absolute Shrinkage and Selection Operator (LASSO), the elastic net (ENET), gradient boosted machines (GBM), a simple average of all forecasts (Comb) and the naïve forecast using (A) mean absolute percentage forecast error, (B) root mean squared forecast error, (C) mean absolute forecast error and (D) mean absolute scaled error across the forecast horizons of 1–8 weeks ahead].

The relative forecast accuracy for each model was assessed by dividing the dataset first into training (2012–2018) and forecast sets (2018–2022) and comparing the discrepancy between forecasted and observed values. In general, forecasts had more error as the time horizon increased (Fig 2A–2D) with different rates of deterioration across forecast models. Across the forecast set in all model assessment criteria, we found that the naïve forecast performed best in the forecast horizon of 1 to 3 weeks ahead (Fig 2A–2D). Following that, gradient boosted machines (GBM) demonstrated superior forecast performance compared to the naïve forecast and machine learning models which incorporated regularization. Correspondingly, we see that the mean absolute scaled errors for all models were higher than 1 for the forecast horizons of 1–3. However, the Least Absolute Shrinkage and Selection Operator (LASSO), elastic net, GBM and forecast combinations had MASEs which gradually decreased below the threshold of 1 from the forecast horizon of 4 to 8 weeks (Fig 2D).

**Fig 3:**
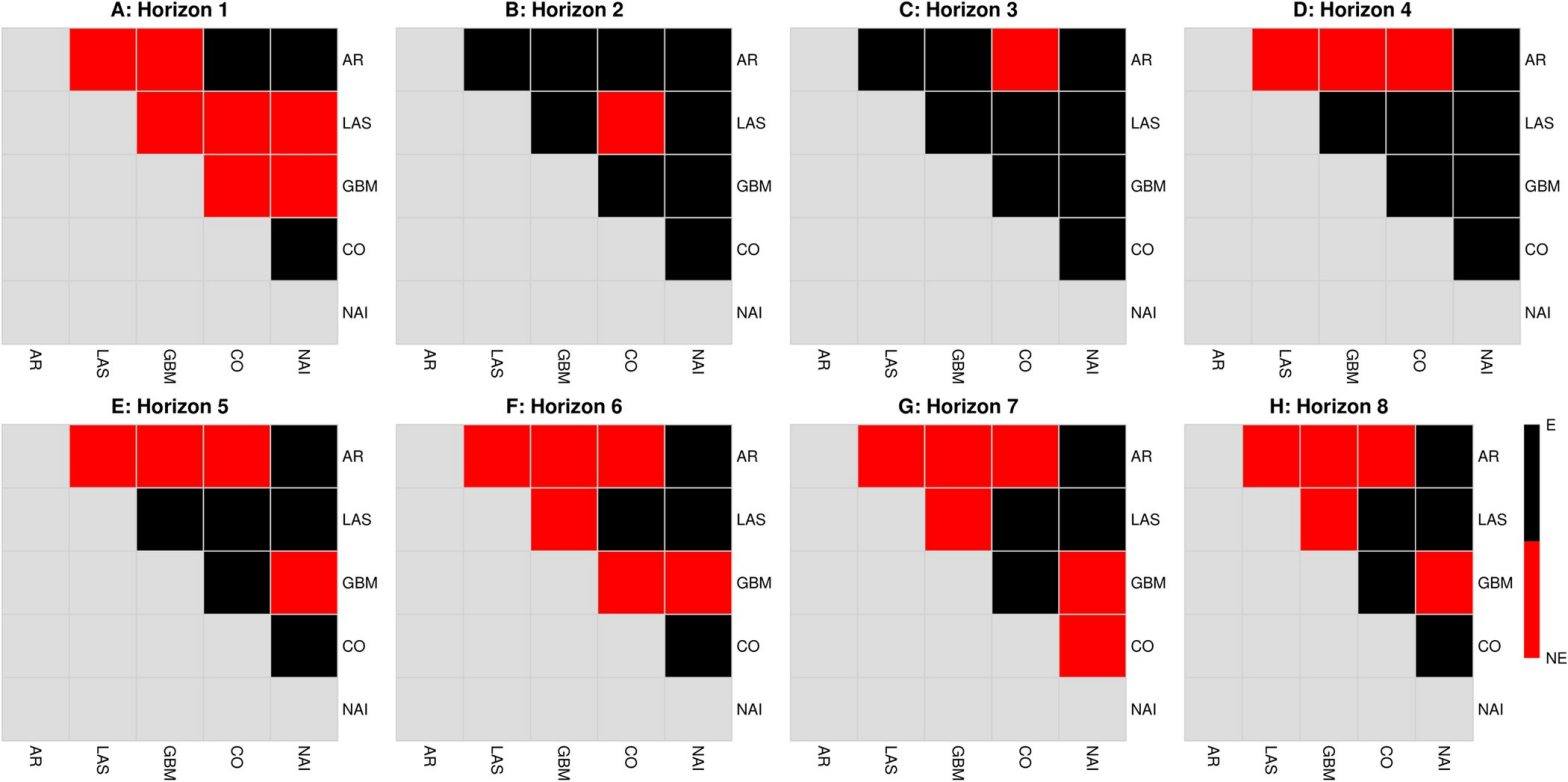
Visualization of Diebold-Mariano (DM) test statistic to test statistical equivalence of forecast errors across models and forecast horizons. Different panels represent the equivalence (E) in black or non-equivalence (NE) in red of forecasts for a specific horizon. This was computed for forecast residuals in the full forecasting dataset in the baseline autoregressive (AR) model, the Least Absolute Shrinkage and Selection Operator (LAS), gradient boosted machines (GBM), a simple average of all forecasts (CO) and the naïve forecast (NAI). Elastic net generated the same regularization as Least Absolute Shrinkage and Selection Operator across all horizons and the DM test statistic was not computed].

Hypothesis testing of forecast errors also consequentially demonstrated that forecast errors were not equivalent between models at the 5% significance level across all forecast horizons. In particular, most forecasts performed significantly better compared to the baseline autoregressive model for horizons 4 to 8 (Fig 3D–3H). The GBM model performed significantly better than at least 50% of forecasts generated by other models in the 5 to 8-week ahead forecast horizons (Fig 3E–3H).

### Structural breaks in URTI transmission influence forecast assessments

**Fig 4:**
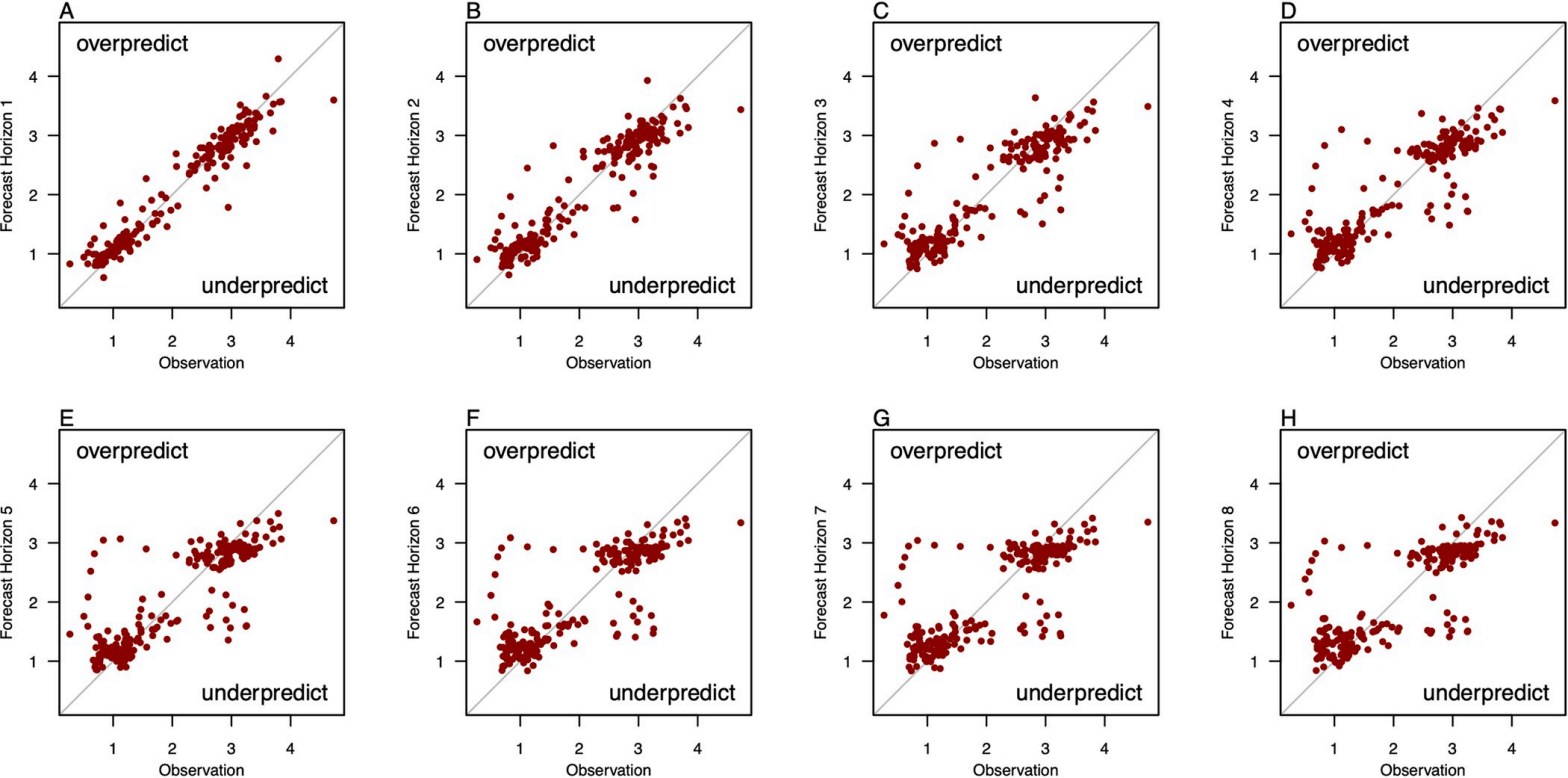
Visualization of forecasts generated using forecast combinations against observations in the full forecasting dataset with the line of equality plotted to ascertain forecasts of over or underprediction. Different panels represent the forecasts versus observations for the forecast horizon of 1–8 steps ahead].

The examination of time series plots showed large discrepancies between forecasts and observations in the period of early 2020–2022. This may be due to structural breaks in disease transmission dynamics attributable to non-pharmaceutical interventions and other phenomena such as changes in health-seeking behaviour motivated by the then-emerging COVID-19 pandemic [23–25]. While across the entire time-series, forecast errors were distributed uniformly across the line of equality, demonstrating that forecasting models are not intentionally biased in magnitude versus observations (Fig 4A–4H), the period of early 2020 had forecasts which overpredicted for around 15 weeks across all forecast horizons (See S1 Text). This has led to inflated, upwardly biased forecast errors for all models, most noticeably in the 2 to 8-weeks ahead horizons (Fig 4B–4H).

**Fig 5:**
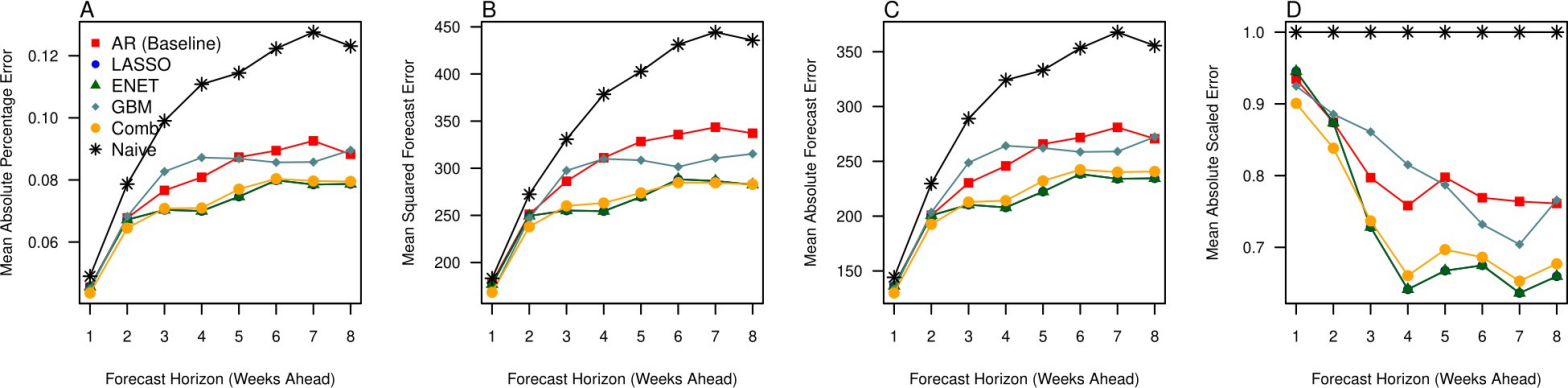
Assessment statistics across the forecast dataset, comparing observations in the period of 2018–2019 to 6 considered forecasting models, which includes the baseline autoregressive (AR) model, the Least Absolute Shrinkage and Selection Operator (LASSO), the elastic net (ENET), gradient boosted machines (GBM), a simple average of all forecasts (Comb) and the naïve forecast using (A) mean absolute percentage forecast error, (B) root mean squared forecast error, (C) mean absolute forecast error and (D) mean absolute scaled error, across the forecast horizons of 1–8 weeks ahead].

Post-hoc assessment of pre-COVID-19 forecasts revealed however, that our forecast models can perform far better on average under more stable transmission conditions from 2018–2019 (Fig 5A–5D) versus transmission conditions where structural breaks were evident in 2020–2022 (Fig 2A–2D). Notably, the mean percentage error for the worst performing model at 8 weeks ahead was less than 15% in 2018–2019 while it was more than 40% in 2020–2022 at 8 weeks ahead (Fig 5A). The forecast performances between models here also showed stark differences between periods. In particular, the LASSO/Elastic-net and the forecast combinations were superior when compared to the other models, and naïve forecasts did not perform better at any forecast horizon in 2018–2019 versus using the full dataset (Figs 2A–2D and 5A–5D).

The results support the use of methods employing regularization to construct forecasts in stable periods of transmission (Figs 2A–2D and 5A–5D), while ensemble methods were able to perform better in conditions where structural breaks were present (Fig 2A–2D). Notably, we see that MAPE degrades slower for the GBM versus other models, being relatively constant at around 8–10% for the period of 2018–2019 and 20–30% for the period of the entire dataset. Considering all forecast assessments, forecast combinations may also be employed conservatively as they have acceptable forecast errors while combining the benefits of both regularization and ensemble methods (Figs 2A–2D and 5A–5D). Forecast combinations uniformly provided more consistent forecasts across all time horizons, across periods of stable transmission and periods of transmission with structural breaks, and had the second-best performance in terms of forecast error rates. Forecast combinations also provide forecasts which are statistically equivalent to GBM in both the full dataset asides for the 6-week ahead forecast (Fig 3A–3H) and to the LASSO/Elastic net the period of 2018–2019 (See S1 Text).

### Impact of environmental variables on forecasts and disease transmission dynamics

While the elastic-net was initially proposed to alleviate multi-collinearity between the disease and environmental predictors of URTI before forecasts were made, these produced identical results as LASSO after parameter tuning using cross-validation (See Figs 2 and 5). Therefore, understanding the effects of meteorological variables on disease case counts relied solely on LASSO to select important explanatory variables to URTI attendances in the full dataset and the linear model to obtain confidence intervals for each regression coefficient through post-selection inference. Over the 8 forecasting sub models, more than 900 environmental predictors were considered with less than 100 selected in determining forecasts over the different forecasting windows (See S1 Text for the full regression output). We thus only describe the general pattern of how environmental variables affect URTI attendance forecasts.

Overall, increases in past mean temperature decreased forward URTI attendances but the regression coefficients were only significant at the 5% level for the 1 to 3-week ahead forecasting window. Increases in past mean total precipitation increased forecasted URTI attendances but the regression coefficients were only significant at the 2-week ahead sub model. Increases in mean relative humidity at shorter forecasting windows (1–4 weeks ahead) were associated with lower URTI attendances but did not affect forecasts at 5–8 weeks ahead. Mean absolute humidity did not affect forecasts at 1–3 weeks ahead but increases in absolute humidity at longer forecast windows for 4–8 weeks ahead were associated with lower forecasted URTI attendances.

## Discussion

Our results demonstrate that across the full dataset, gradient boosted machines (GBM) worked best across the 4 to 5-week ahead forecast horizons (Fig 2A–2D) while the naïve forecast performed better in the 1 to 3-week ahead forecast (Fig 3A–3H). In contrast, the Least Absolute Shrinkage and Selection Operator (LASSO) and Elastic-net performed better throughout the stable transmission period (Fig 5A–5D). However, for both the full and subsetted datasets, GBM and LASSO forecasts were statistically equivalent to the forecast combinations, which was a simple average of all forecasts for that week ahead, and provided the second-best forecast across all time horizons. Simple forecast combinations can be used to incorporate the benefits of both machine learning models and provide consistent forecasts for different periods of transmission, and across other scenarios, can provide good forecasts for diseases such as influenza [16] and dengue [26].

Our approach provides relatively good forecasts with high accuracy up to 8 weeks ahead in stable periods of transmission with the error range at ~10% for the best performing models (Fig 5A). We improve over previous forecasting models [12,16,27] by first incorporating a high-dimensional set of disease and environmental variables which can co-move with polyclinic attendances, and accounted for this variable set by using well-established methodologies to accommodate for many variables. These methods are also increasingly important in the public health domain where datasets are becoming increasingly large. In particular, several benefits are attributable to these tools. Firstly, LASSO conducts rapid selection of predictors, tuned using cross-validation approaches on the training data, which helps to optimize in-sample predictive performance, whereas GBM optimizes the model by iteratively improving over weak predictors, making the models selected with the optimal tuning parameter well suited for out-of-sample forecasting. Additionally, both tools allow for a large set of environmental and disease predictors to be considered. Non-predictive variables obtain zero coefficients at the optimal values of the penalty term in cross-validation and thereby drop out of the final model for LASSO. GBM can also iteratively reduce the prediction variance generated from many weak predictors through an ensemble approach. Next, the incorporation of environmental variables restricts models to direct, rather than recursive forecasts, which may be more suited to diseases where transmission is almost always endogenously defined. This means that distinct sub models with parameters tuned to maximize predictive accuracy over that time-horizon are used, thereby providing superior forecasts versus baseline models. Furthermore, standard tools used for recursive forecasting tend to mean-revert, which means that their performance may deteriorate much faster over larger time horizons in periods of structural breaks. Lastly, forecast combination combine LASSO/GBM benefits and may be a viable alternative for forecasting over many transmission scenarios, as evident from structural breaks in the data from 2020 onwards (See S1 Text).

While the interpretation of coefficients was difficult due to the large number of predictors and lags selected to train models at each forecasting window, using LASSO and post-selection inference, we found relatively good concordance with current literature on the impact of environmental variables on URTIs. In particular, past studies have shown, similar to our coefficient estimates, that higher relative and absolute humidity were associated to lower URTI burdens. A possible explanation, as suggested previously, was that in relatively higher humidity, removal of infectious particles is favored by both increasing the settling of large, water-laden droplets and by hastening virus inactivation, thereby attenuating respiratory virus transmission [28]. Finer-scale epidemiological studies should be done to confirm these explanations as the dataset used for this study was national, rather than on the individual level.

There were however, several limitations to our approach. Firstly, although ensemble methods such as GBM were able to perform acceptably even in periods where structural breaks are present, yielding an error rate of less than 25% in 8 weeks ahead forecasts (Fig 2A) in the emerging phase of COVID-19 where severe non-pharmaceutical interventions (NPIs) were implemented, all considered models generated overpredictions over the course of 15 weeks. Forecasts here were inherently generated on past stochastic behavior, calibrated on past observations, which can perform poorly in periods where structural breaks are evident. This highlights the need to interpret forecasts with both epidemiological information and public health policy knowledge. Secondly, while global data sources are now also available on NPIs implemented due to COVID from 2020–2022, this cannot be trained over the entire history where URTI attendances are collected (2012–2022), which means that NPI effects pre-2020 can only be evaluated ex-post and accounted for using simulation or transmission dynamic approaches rather than the statistical tools proposed herewith. Thirdly, the interpretation of coefficients were very difficult due to the large number of predictors and lags selected to train models at each forecasting window. Fourthly, GBM is an ensemble method combining a large number of weak predictive models where one of the only means to interpret predictors is to look at variable importance. Variable importance provides a notion of how much predictors determine the predictions, and cannot provide us with information on how a specific predictor influences the disease of interest–GBM therefore provides output which has sparser epidemiological/ecological interpretation. Therefore, while the complexity is needed to accommodate for the environmental and social factors determining URTI attendances in good and robust forecasts, our tools need to be combined with standard models to appropriately identify factors leading to increased URTI attendances. This is beyond the scope of this paper. Lastly, parameter tuning rather than model training using GBM is computationally expensive. Within the training dataset, grid searching and re-running cross validation across many parameters over rolling forecasting window is required, which was why default parameters were used in the GBM tuning stage. We however demonstrate that the forecasts generated were able to perform well despite this limitation. Therefore, there is considerable potential to improve the ensemble forecasts presented here through using well-calibrated GBM parameters but would require an extensive grid-search and computational resources to confirm.

## Conclusion

The proposed methods can be used for outbreak preparedness and guide healthcare resource planning. In particular, forecast combinations combine the benefits of many forecasts and perform consistently in both stable transmission periods and periods where structural breaks are evident in data.

## Supporting information

S1 Text Appendix containing additional details on results. (DOCX)
