# Supplementary material for: Forecasting upper respiratory tract infection burden using high-dimensional time series data and forecast combinations
Source: PLoS Comput Biol. 2023 Feb 7;19(2):e1010892. doi: 10.1371/journal.pcbi.1010892 (PMC9983836; doi:10.1371/journal.pcbi.1010892)
Supplement: S1 Text — (DOCX) [file pcbi.1010892.s001.docx]

**Supplementary Information**

[Figure 1 in S1 File: Visualization of Diebold-Mariano (DM) test statistic to test statistical equivalence of forecast errors across models and forecast horizons at the 10% level. Different panels represent the statistical equivalence (E) in black or non-equivalence (NE) in red of forecasts for a specific horizon for the period before 2020. This was computed for forecast residuals in the full forecasting dataset in the baseline autoregressive (AR) model, the Least Absolute Shrinkage and Selection Operator (LAS), gradient boosted machines (GBM), a simple average of all forecasts (CO) and the naïve forecast (NAI). Elastic net generated the same regularization as Least Absolute Shrinkage and Selection Operator across all horizons and the DM test statistic was not computed.]

| Predictive Variable | Resolution | Source |
| --- | --- | --- |
| Acute Conjunctivitis | Epidemiological Week | Infectious Disease Bulletin, Singapore |
| Acute Diarrhoea | Epidemiological Week | Infectious Disease Bulletin, Singapore |
| Acute Upper Respiratory Tract infections | Epidemiological Week | Infectious Disease Bulletin, Singapore |
| Acute Viral Hepatitis A | Epidemiological Week | Infectious Disease Bulletin, Singapore |
| Acute Viral Hepatitis B | Epidemiological Week | Infectious Disease Bulletin, Singapore |
| Acute Viral Hepatitis C | Epidemiological Week | Infectious Disease Bulletin, Singapore |
| Acute Viral Hepatitis E | Epidemiological Week | Infectious Disease Bulletin, Singapore |
| Campylobacter enteritis | Epidemiological Week | Infectious Disease Bulletin, Singapore |
| Chickenpox | Epidemiological Week | Infectious Disease Bulletin, Singapore |
| Dengue Fever | Epidemiological Week | Infectious Disease Bulletin, Singapore |
| Dengue Haemorrhagic Fever | Epidemiological Week | Infectious Disease Bulletin, Singapore |
| HFMD | Epidemiological Week | Infectious Disease Bulletin, Singapore |
| Legionellosis | Epidemiological Week | Infectious Disease Bulletin, Singapore |
| Measles | Epidemiological Week | Infectious Disease Bulletin, Singapore |
| Melioidosis | Epidemiological Week | Infectious Disease Bulletin, Singapore |
| Meningococcal Infection | Epidemiological Week | Infectious Disease Bulletin, Singapore |
| Mumps | Epidemiological Week | Infectious Disease Bulletin, Singapore |
| Paratyphoid | Epidemiological Week | Infectious Disease Bulletin, Singapore |
| Pneumococcal Disease invasive | Epidemiological Week | Infectious Disease Bulletin, Singapore |
| Salmonellosis non enteric fevers | Epidemiological Week | Infectious Disease Bulletin, Singapore |
| Typhoid | Epidemiological Week | Infectious Disease Bulletin, Singapore |
| Mean temperature | Epidemiological Week | ERA-5 Land |
| Mean total precipitation | Epidemiological Week | ERA-5 Land |
| Mean absolute humidity | Epidemiological Week | Derived |
| Mean relative humidity | Epidemiological Week | Derived |
| Mean leaf index | Epidemiological Week | ERA-5 Land |
| Minimum temperature | Epidemiological Week | ERA-5 Land |
| Minimum total precipitation | Epidemiological Week | ERA-5 Land |
| Minimum absolute humidity | Epidemiological Week | Derived |
| Minimum relative humidity | Epidemiological Week | Derived |
| Minimum leaf index | Epidemiological Week | ERA-5 Land |
| Maximum temperature | Epidemiological Week | ERA-5 Land |
| Maximum total precipitation | Epidemiological Week | ERA-5 Land |
| Maximum absolute humidity | Epidemiological Week | Derived |
| Maximum relative humidity | Epidemiological Week | Derived |
| Maximum leaf index | Epidemiological Week | ERA-5 Land |

[Table 1 in S1 File: list of data used as predictive variables for 1-8 week ahead URTI forecasts]

[Figure 2 in S1 File: Visualization of URTI case counts versus one-week ahead forecasts from the autoregressive model with dependent variable being past URTI case counts, with number of lags selected through backward stepwise selection and Akaike Information Criterion as the selection criterion.]

[Figure 3 in S1 File: Visualization of URTI case counts versus two-week ahead forecasts from the autoregressive model with dependent variable being past URTI case counts, with number of lags selected through backward stepwise selection and Akaike Information Criterion as the selection criterion.]

[Figure 4 in S1 File: Visualization of URTI case counts versus three-week ahead forecasts from the autoregressive model with dependent variable being past URTI case counts, with number of lags selected through backward stepwise selection and Akaike Information Criterion as the selection criterion.]

[Figure 5 in S1 File: Visualization of URTI case counts versus four-week ahead forecasts from the autoregressive model with dependent variable being past URTI case counts, with number of lags selected through backward stepwise selection and Akaike Information Criterion as the selection criterion.]

[Figure 6 in S1 File: Visualization of URTI case counts versus five-week ahead forecasts from the autoregressive model with dependent variable being past URTI case counts, with number of lags selected through backward stepwise selection and Akaike Information Criterion as the selection criterion.]

[Figure 7 in S1 File: Visualization of URTI case counts versus six-week ahead forecasts from the autoregressive model with dependent variable being past URTI case counts, with number of lags selected through backward stepwise selection and Akaike Information Criterion as the selection criterion.]

[Figure 8 in S1 File: Visualization of URTI case counts versus seven-week ahead forecasts from the autoregressive model with dependent variable being past URTI case counts, with number of lags selected through backward stepwise selection and Akaike Information Criterion as the selection criterion.]

[Figure 9 in S1 File: Visualization of URTI case counts versus eight-week ahead forecasts from the autoregressive model with dependent variable being past URTI case counts, with number of lags selected through backward stepwise selection and Akaike Information Criterion as the selection criterion.]

****[Figure 10 in S1 File: Visualization of URTI case counts versus one-week ahead forecasts from gradient boosted machines, using all applicable diseases of interest in the infectious disease bulletin and environmental covariates of up to 8 weeks lag as predictors]

****[Figure 11 in S1 File: Visualization of URTI case counts versus two-week ahead forecasts from gradient boosted machines, using all applicable diseases of interest in the infectious disease bulletin and environmental covariates of up to 8 weeks lag as predictors]**** [Figure 12 in S1 File: Visualization of URTI case counts versus three-week ahead forecasts from gradient boosted machines, using all applicable diseases of interest in the infectious disease bulletin and environmental covariates of up to 8 weeks lag as predictors]**** [Figure 13 in S1 File: Visualization of URTI case counts versus four-week ahead forecasts from gradient boosted machines, using all applicable diseases of interest in the infectious disease bulletin and environmental covariates of up to 8 weeks lag as predictors]**** [Figure 14 in S1 File: Visualization of URTI case counts versus five-week ahead forecasts from gradient boosted machines, using all applicable diseases of interest in the infectious disease bulletin and environmental covariates of up to 8 weeks lag as predictors]**** [Figure 15 in S1 File: Visualization of URTI case counts versus six-week ahead forecasts from gradient boosted machines, using all applicable diseases of interest in the infectious disease bulletin and environmental covariates of up to 8 weeks lag as predictors]**** [Figure 16 in S1 File: Visualization of URTI case counts versus seven-week ahead forecasts from gradient boosted machines, using all applicable diseases of interest in the infectious disease bulletin and environmental covariates of up to 8 weeks lag as predictors]**** [Figure 17 in S1 File: Visualization of URTI case counts versus eight-week ahead forecasts from gradient boosted machines, using all applicable diseases of interest in the infectious disease bulletin and environmental covariates of up to 8 weeks lag as predictors]**** [Figure 18 in S1 File: Visualization of URTI case counts versus one-week ahead forecasts from the Least Absolute Shrinkage and Selection Operator, using all applicable diseases of interest in the infectious disease bulletin and environmental covariates of up to 8 weeks lag as predictors]

****[Figure 19 in S1 File: Visualization of URTI case counts versus two-week ahead forecasts from the Least Absolute Shrinkage and Selection Operator, using all applicable diseases of interest in the infectious disease bulletin and environmental covariates of up to 8 weeks lag as predictors]

**** [Figure 20 in S1 File: Visualization of URTI case counts versus three-week ahead forecasts from the Least Absolute Shrinkage and Selection Operator, using all applicable diseases of interest in the infectious disease bulletin and environmental covariates of up to 8 weeks lag as predictors]

**** [Figure 21 in S1 File: Visualization of URTI case counts versus four-week ahead forecasts from the Least Absolute Shrinkage and Selection Operator, using all applicable diseases of interest in the infectious disease bulletin and environmental covariates of up to 8 weeks lag as predictors]

**** [Figure 22 in S1 File: Visualization of URTI case counts versus five-week ahead forecasts from the Least Absolute Shrinkage and Selection Operator, using all applicable diseases of interest in the infectious disease bulletin and environmental covariates of up to 8 weeks lag as predictors]

**** [Figure 23 in S1 File: Visualization of URTI case counts versus six-week ahead forecasts from the Least Absolute Shrinkage and Selection Operator, using all applicable diseases of interest in the infectious disease bulletin and environmental covariates of up to 8 weeks lag as predictors]

**** [Figure 24 in S1 File: Visualization of URTI case counts versus seven-week ahead forecasts from the Least Absolute Shrinkage and Selection Operator, using all applicable diseases of interest in the infectious disease bulletin and environmental covariates of up to 8 weeks lag as predictors]

**** [Figure 25 in S1 File: Visualization of URTI case counts versus eight-week ahead forecasts from the Least Absolute Shrinkage and Selection Operator, using all applicable diseases of interest in the infectious disease bulletin and environmental covariates of up to 8 weeks lag as predictors]

****[Figure 26 in S1 File: Visualization of URTI case counts versus one-week ahead forecasts from forecast combination (simple average of all forecasts except the naïve forecast)]

****[Figure 27 in S1 File: Visualization of URTI case counts versus two-week ahead forecasts from forecast combination (simple average of all forecasts except the naïve forecast)]

****[Figure 28 in S1 File: Visualization of URTI case counts versus three-week ahead forecasts from forecast combination (simple average of all forecasts except the naïve forecast)]

****[Figure 29 in S1 File: Visualization of URTI case counts versus four-week ahead forecasts from forecast combination (simple average of all forecasts except the naïve forecast)]

****[Figure 30 in S1 File: Visualization of URTI case counts versus five-week ahead forecasts from forecast combination (simple average of all forecasts except the naïve forecast)]

****[Figure 31 in S1 File: Visualization of URTI case counts versus six-week ahead forecasts from forecast combination (simple average of all forecasts except the naïve forecast)]

****[Figure 32 in S1 File: Visualization of URTI case counts versus seven-week ahead forecasts from forecast combination (simple average of all forecasts except the naïve forecast)]

****[Figure 33 in S1 File: Visualization of URTI case counts versus eight-week ahead forecasts from forecast combination (simple average of all forecasts except the naïve forecast)]

**** [Figure 34 in S1 File: Visualization of URTI case counts versus one-week ahead naïve forecasts (taking the latest available observation of URTI case counts at the contemporaneous time point as the one-step ahead forecast]

****[Figure 35 in S1 File: Visualization of URTI case counts versus two-week ahead naïve forecasts (taking the latest available observation of URTI case counts at the contemporaneous time point as the one-step ahead forecast]

**** [Figure 36 in S1 File: Visualization of URTI case counts versus three-week ahead naïve forecasts (taking the latest available observation of URTI case counts at the contemporaneous time point as the one-step ahead forecast]

**** [Figure 36 in S1 File: Visualization of URTI case counts versus four-week ahead naïve forecasts (taking the latest available observation of URTI case counts at the contemporaneous time point as the one-step ahead forecast]

**** [Figure 37 in S1 File: Visualization of URTI case counts versus five-week ahead naïve forecasts (taking the latest available observation of URTI case counts at the contemporaneous time point as the one-step ahead forecast]

**** [Figure 38 in S1 File: Visualization of URTI case counts versus six-week ahead naïve forecasts (taking the latest available observation of URTI case counts at the contemporaneous time point as the one-step ahead forecast]**** [Figure 39 in S1 File: Visualization of URTI case counts versus seven-week ahead naïve forecasts (taking the latest available observation of URTI case counts at the contemporaneous time point as the one-step ahead forecast]

**** [Figure 40 in S1 File: Visualization of URTI case counts versus eight-week ahead naïve forecasts (taking the latest available observation of URTI case counts at the contemporaneous time point as the one-step ahead forecast
